# Medical Cannabis in Children

**DOI:** 10.5041/RMMJ.10386

**Published:** 2020-01-30

**Authors:** Adi Aran, Dalit Cayam-Rand

**Affiliations:** Neuropediatric Unit, Shaare Zedek Medical Center, Jerusalem, Israel

**Keywords:** Autism, cannabis, CBD, children, epilepsy, THC

## Abstract

The use of medical cannabis in children is rapidly growing. While robust evidence currently exists only for pure cannabidiol (CBD) to treat specific types of refractory epilepsy, in most cases, artisanal strains of CBD-rich medical cannabis are being used to treat children with various types of refractory epilepsy or irritability associated with autism spectrum disorder (ASD). Other common pediatric disorders that are being considered for cannabis treatment are Tourette syndrome and spasticity. As recreational cannabis use during youth is associated with serious adverse events and medical cannabis use is believed to have a relatively high placebo effect, decisions to use medical cannabis during childhood and adolescence should be made with caution and based on evidence. This review summarizes the current evidence for safety, tolerability, and efficacy of medical cannabis in children with epilepsy and in children with ASD. The main risks associated with use of Δ9-tetrahydrocannabinol (THC) and CBD in the pediatric population are described, as well as the debate regarding the use of whole-plant extract to retain a possible “entourage effect” as opposed to pure cannabinoids that are more standardized and reproducible.

## NEUROACTIVITY OF CANNABIS

The cannabis plant has a substantial effect on social behavior in humans.^1^ It enhances interpersonal communication^2^ and decreases hostile feelings.^3^ Similar to other plants, cannabis contains hundreds of compounds, including terpenes and flavonoids, many of which have a known or presumed neurological effect.^4^ Cannabis also contains over a hundred unique compounds called phytocannabinoids (plant-derived cannabinoids).

The two main phytocannabinoids are cannabidiol (CBD) and Δ9-tetrahydrocannabinol (THC). These compounds were characterized in 1963 and 1964, respectively, by Professor Raphael Mechoulam and colleagues from Israel.^5^,^6^ Mechoulam found that THC is the plant’s main psychoactive component, responsible for the feeling of a “high.” This effect in the brain is mediated by an abundant G-protein-coupled receptor, which he named cannabinoid type 1 receptor (CB_1_R). A second receptor that is also directly activated by THC was isolated later from macrophages in the spleen and was named cannabinoid type-2 receptor (CB_2_R).^7^ Accordingly, the main effect mediated by CB_2_R is immunomodulation. This receptor is not significantly expressed in the brain under normal conditions but can be found on glial cells in various brain pathologies.

The two main endogenous ligands of the cannabinoid receptors (“endocannabinoids”) are N-arachidonoylethanolamine (AEA or anandamide) and 2-arachidonoylglycerol (2-AG).

**Figure 1:**
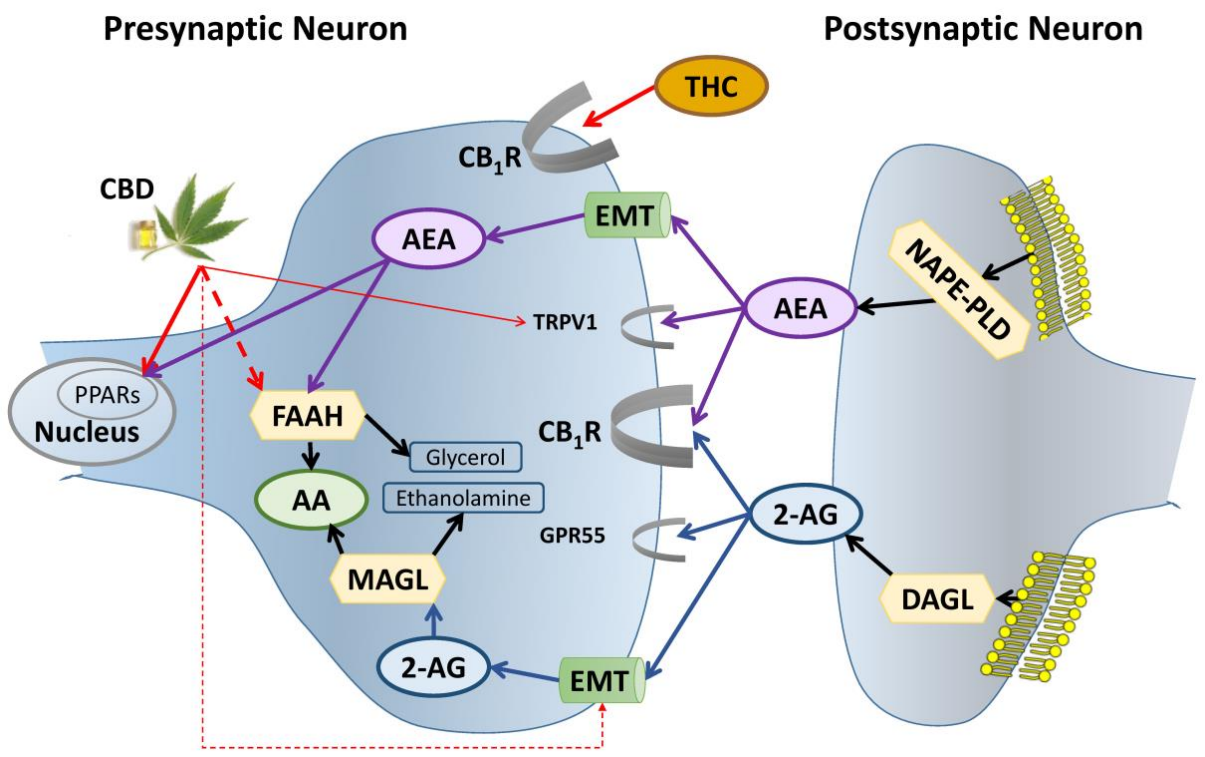
Biosynthesis, Degradation, and Receptor Binding of AEA and 2-AG. AEA is synthesized from membrane phospholipids in the postsynaptic neuron by NAPE-PLD. It crosses the synapse “against the traffic” and activates CB_1_R and TRPV1 on the presynaptic neuron. Following reuptake to the presynaptic neuron by the EMT, AEA activates nuclear receptors—PPARs—and is degraded by FAAH. THC directly activates CB_1_R; CBD inhibits FAAH and EMT (increasing AEA levels), the endogenous ligand of CB_1_R. Like AEA, CBD activates PPARs and TRPV1. 2-AG, 2-arachidonoylglycerol (blue ellipses and related lines); AA, arachidonic acid (green ellipses); AEA, anandamide (purple ellipses and related lines); CB_1_R, cannabinoid type 1 receptor; CBD, cannabidiol; DAGL, diacylglycerol lipase; EMT, endocannabinoid membrane transporter (green tubes); FAAH, fatty acid amide hydrolase; GPR55, G protein-coupled receptor 55; MAGL, monoacylglycerol lipase; NAPE-PLD, N-acylphosphatidylethanolamine-specific phospholipase D; PPARs, peroxisome proliferator-activated receptors; THC, Δ9-tetrahydrocannabinol; TRPV1, transient receptor potential channels of vanilloid type-1. Broken lines = inhibition; half ellipses ( 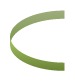) = receptors; hexagons = enzymes; yellow mesh = cell membrane. Thicker lines and half ellipses are of greater importance than the thinner ones. Black lines, other pathways; red lines, phytocannabinoid pathways.

Figure 1 demonstrates the endocannabinoid system. Endocannabinoids are produced “on demand” in postsynaptic neurons and act as retrograde signaling messengers in overactive brain circuits. By activating CB_1_R in presynaptic neurons, they modulate the synaptic release of neurotransmitters into the synaptic cleft and attenuate the synaptic activity in that circuit.^8^ After reuptake of the endocannabinoids to the presynaptic neuron by the endocannabinoid membrane transporter, they are immediately hydrolyzed.

While THC directly activates the endocannabinoid system through CB_1_R, CBD does not activate CB_1_R directly and is not psychoactive.^9^ Cannabidiol has a relatively high toxicity threshold and appears to have anxiolytic, antipsychotic, and neuroprotective properties that may be mediated through receptors such as serotonin 5-HT1A, glycine α3 and α1, TRPV1, GPR55, and PPARγ, and by inhibiting adenosine reuptake.^10^–^13^ Cannabidiol also inhibits the enzyme fatty acid amide hydrolase (FAAH) that degrades anandamide, the endogenous ligand of CB_1_R, and hence can indirectly activate the endocannabinoid system (Figure 1).

While 99% pure cannabinoids are more reproducible and standardized than whole-plant extract and thus preferred as a study drug, several studies suggest a synergistic effect for the numerous cannabis compounds in the whole-plant extract.^4^,^14^–^19^ This so-called “entourage effect” remains controversial.^20^

Future studies should directly assess the effects of pure cannabinoids versus whole-plant extracts in various disorders among different target populations.

## MAIN RISKS OF CANNABIS IN CHILDREN

The current knowledge on the long-term side-effects of cannabinoids is based mainly on longitudinal follow-up of recreational cannabis users.^21^–^23^ Several large studies have demonstrated that the main risks of decreased motivation,^24^–^26^ addiction,^27^ mild cognitive decline,^23^,^25^,^28^,^29^ and schizophrenia^25^,^30^–^32^ are directly related to the THC and CBD concentrations in the strain used,^33^ i.e. the higher the ratio of THC to CBD, the greater the risk. The risk is also elevated among those with younger onset of use (<18 years) and in the presence of other risk factors, such as a family history of schizophrenia and concomitant use of alcohol and tobacco.^33^,^34^ Notably, these studies contained very few participants under 10 years old and did not assess daily use of medical cannabis.

Longitudinal follow-up studies in children with epilepsy receiving pure CBD suggest high tolerability and safety,^35^–^37^ but these studies included very few participants younger than 5 years old.

Animal studies suggest that using pure CBD and its analogue cannabidivarin (CBDV) during early development is relatively safe^38^,^39^ while the use of THC, with or without CBD, during early development was found to impair brain structure and function.^40^–^43^

Short-term adverse events of pure CBD or CBD-rich whole-plant extracts include somnolence, weight loss, and increased liver transaminases.^35^,^36^,^44^–^47^

## MEDICAL CANNABIS FOR CHILDREN WITH EPILEPSY

Epilepsy is a common neurological disorder, affecting 0.5%–1% of the world’s population.^48^ Despite the availability of many effective antiepileptic drugs, about one-third of epileptic patients will continue to have treatment-refractory seizures.^49^ If a patient continues to have seizures despite appropriate treatment trials with three medications, the probability of achieving seizure freedom with subsequent medications is less than 3.5%.^49^ In such cases, treatment options include epileptic surgery, vagal nerve stimulation, or ketogenic diet. However, for the numerous patients who are not eligible for surgery or do not respond to these treatments, medical cannabis may offer more hope for seizure reduction compared with other pharmacological interventions.

Cannabis treatment for seizures has a long history; it has been used as an anticonvulsant in the ancient Middle East and India for at least 4000 years.^50^ More recently, leading nineteenth-century neurologists Sir John Russell Reynolds and Sir William Richard Gowers sporadically used THC-rich cannabis to treat seizures. The use of cannabis for epilepsy gradually ceased following the introduction of phenobarbitone in 1912 and phenytoin in 1937. Small studies, mainly of THC-rich cannabis for children with epilepsy, re-emerged in the 1970s with mixed results. Following the discovery of the endocannabinoid system in the 1990s and its major role in neuromodulation, including the attenuation of overactive brain circuits, studies in animal models and anecdotes of successful treatment in refractory epilepsy cases began to accumulate. However, larger-scale clinical studies of cannabinoids in epilepsy were only conducted in recent years.^50^ These studies focus on the safer cannabinoid, CBD, which seems to have more of an antiepileptic effect than THC in preclinical studies.

A plant-derived pure CBD compound (brand name: Epidiolex) was approved by the US Food and Drug Administration (FDA) in 2018 for treating two severe forms of epilepsy—Dravet and Lennox–Gastaut syndromes^51^—following a series of successful safety and efficacy studies.^36^,^52^–^54^

A recent systematic review and meta-analysis of the efficacy and tolerability of pure CBD and CBD-rich medical cannabis revealed that CBD is more effective than placebo for treatment-resistant epilepsy, regardless of the etiology of the epileptic syndrome.^37^ Adverse events included somnolence, decreased appetite and weight, irritability, increased seizure frequency, and diarrhea (in some of the studies). Laboratory abnormalities included elevation of liver transaminases in patients who also received valproic acid. Short-term adverse events were found to be similar for pure CBD and CBD-rich medical cannabis. Adverse events were more frequent at treatment onset compared with long-term follow-up.^37^ Clinicians should also be aware of cross-reactivity between CBD or CBD-rich medical cannabis and antiepileptic medications that are also metabolized by the cytochrome P450 complex. Notably, active metabolites of benzodiazepines significantly increase with concomitant use of CBD and CBD-rich medical cannabis.

## MEDICAL CANNABIS FOR CHILDREN WITH AUTISM SPECTRUM DISORDER

Autism spectrum disorder (ASD) affects up to 2.5% of children worldwide and is a major public health challenge.^55^ Individuals with ASD have social and communication difficulties, stereotyped or repetitive behaviors and interests, sensory problems, and, in many cases, cognitive impairment and disruptive behaviors. These deficits are present in early childhood and lead to significant disability.^56^

Approximately 50% of children and adolescents with ASD demonstrate behavioral difficulties, including tantrums, non-compliance, aggression, and self-injury.^57^–^59^ This rate is higher than in any other neurodevelopmental disorder.^60^–^65^ The behavioral difficulties of children with ASD increase their social isolation^66^,^67^ and often cause more distress to caregivers than the core autistic symptoms.^68^–^70^ Maladaptive behaviors also limit the child’s ability to benefit from intervention efforts, thereby impairing the long-term prognosis.^71^

Standard treatment for these problems is based on behavioral interventions^71^–^75^ combined with medications,^76^,^77^ particularly atypical antipsychotics^78^–^84^ and mood stabilizers.^85^ However, both the efficacy and tolerability of pharmacotherapy in children with ASD are less favorable than among typically developing children with similar symptoms.^86^

As a result, despite extensive behavioral and medical treatment, 40% of the children and youth with ASD suffer from maladaptive behavior^87^ that severely impacts their quality of life and takes a heavy toll on their families.^69^,^88^ The frustration from current medical treatment leads to an exceptionally high percentage of parents seeking help from complementary and alternative medicine.^89^,^90^

Epilepsy is one of the most common comorbid conditions in ASD, affecting 10%–30% of children and youth with ASD,^91^ and several pathophysiological processes are implicated in both disease processes.^92^ Hence, the positive effect of cannabinoids in refractory patients is relevant for individuals with ASD.

The CB_1_R is highly expressed in the frontal cortex and subcortical areas associated with social functioning.^93^,^94^ The CB1 receptors and their endogenous ligands anandamide and 2-AG regulate social play and social anxiety in animal models^95^–^100^ and in humans.^101^–^103^

Activation of the endocannabinoid system in the nucleus accumbens (anandamide mobilization and consequent activation of CB1 receptors) driven by oxytocin, a neuropeptide that reinforces social bonding, was demonstrated to be necessary and sufficient to express the rewarding properties of social interaction.^104^ Reduced endocannabinoid activity was demonstrated in several animal models of ASD,^105^ including monogenic (fragile-X,^106^ neuroligin 3^107^), polygenic (BTBR^105^), and environmental (rat valproic acid^108^) models. Activation of the endocannabinoid system^105^–^108^ and administration of CBD^105^ have been shown to restore social deficits in some models. A single oral administration of 600 mg CBD to 34 men (17 neurotypicals and 17 with ASD) increased prefrontal gamma-aminobutyric acid (GABA) activity in neurotypicals and decreased GABA activity in those with ASD.^109^ Additionally, children with ASD have been found to have lower peripheral endocannabinoid levels.^110^,^111^

Therefore, dysregulation of the endocannabinoid system may play an important role in ASD pathophysiology and may represent a target for pharmacological intervention.^112^

Four uncontrolled case-series, including 60, 188, 53, and 18 children with ASD and severe behavioral problems, reported high tolerability and efficacy of artisanal CBD-rich cannabis strains.^44^–^47^ In the largest cohort, data collection was partial, and there was also an unknown overlap between the first three cohorts. Most participants were followed for at least 6 months, and the retention rate was about 80%.

The treatment was reported to substantially decrease the irritability and anxiety in most of the participants and to improve the social deficits in about half of the subjects, but these results should be interpreted cautiously.

Cannabinoid treatment is associated with a relatively high placebo effect, compared with other pharmacological treatments.^113^ Placebo effect is expected to be even higher in ASD studies which use subjective behavioral assessments.^114^ Hence, placebo-controlled studies are required even for a preliminary assessment of efficacy. To date, only one controlled study was completed (NCT02956226) and another is ongoing (NCT03202303).

NCT02956226 was a phase 2, proof-of-concept trial, conducted in a single referral center, Shaare Zedek Medical Center, Jerusalem, Israel. In this double-blind, placebo-controlled trial, 150 children (age 5–21 years) with ASD and behavioral problems (Clinical Global Impression of Severity ≥4), were randomized (in a 1:1:1 ratio) to receive either placebo or one of two cannabinoid solutions for 12 weeks. The cannabinoid solutions were: (1) whole-plant cannabis extract containing cannabidiol (CBD) and Δ9-tetrahydrocannabinol (THC) in a 20:1 ratio; and (2) purified CBD and THC in the same ratio and dose, without other components of the cannabis plant such as minor cannabinoids, terpenes, and flavonoids which may also contribute to the therapeutic effect in an entourage effect.^4^ The rationale for THC use was previous experience with similar cannabis strains in open-label studies^44^–^46^ and the known effects of THC on social behavior.^115^ The taste, smell, and texture of the three study interventions were carefully adjusted for similarity, which was approved by a professional taster. Participants received either placebo or cannabinoids for 12 weeks to test efficacy, followed by a 4-week washout, and crossed-over to receive another treatment for 12 weeks to further assess tolerability. The treatments were given orally as an add-on to any pre-existing treatment, with an average CBD dose of 5.5 mg/kg/day, divided into three daily doses. There were no serious treatment-related adverse events. The most prevalent adverse events were somnolence and loss of appetite. In some of the measures, cannabinoids reduced the irritability and even the core symptoms of autism significantly more than placebo, with no advantage of the whole-plant extract over the pure cannabinoids.

## MEDICAL CANNABIS FOR CHILDREN WITH SPASTICITY AND OTHER INDICATIONS

An open-label study of 25 children (age 1–17 years) with a complex motor disorder^116^ demonstrated improvement in spasticity and dystonia, sleep difficulties, pain severity, and quality of life. The participants received one of two artisanal strains of CBD-rich cannabis for 5 months: either a 20:1 CBD:THC ratio or a 6:1 ratio. No significant differences were found between the two strains. Two case series, one with 12 children with treatment-refractory spasticity related to developmental disorders^117^ and one with 7 children with pantothenate kinase-associated neurodegeneration (PKAN),^118^ reported improvement in the spasticity and dystonia in some of the children after treatment with dronabinol, a synthetic form of THC (spasticity) or various cannabis strains (PKAN). Case reports of children who received cannabis for other indications^119^ included cases of: neuropathic pain and comorbid major depressive disorder (dronabinol, *n*=2), anxiety and sleep in PTSD (CBD, *n*=1), and Tourette syndrome (THC, *n*=1). Larger open-label studies and randomized studies are required prior to clinical use of cannabis for these indications.

## DISCUSSION

Public interest in cannabis-based treatment is rapidly growing, especially in disorders with substantial unmet needs such as pediatric ASD and refractory epilepsy. This review summarizes current knowledge on the neuroactivity of cannabinoids, potential risks, and evidence of efficacy and tolerability in epilepsy and ASD. While most patients receive a variety of artisanal strains, only pure CBD for epilepsy has been rigorously evaluated in controlled trials thus far, with modest but significant improvement in motor seizures and acceptable tolerability. Adverse events included somnolence and reduced appetite. Important interactions with antiepileptic drugs include an increased risk of hepatotoxicity with valproic acid and an increased level of active metabolites with benzodiazepines, contributing to somnolence and potentially to efficacy.

While some studies suggest that artisanal strains with a very high ratio of CBD:THC (e.g. 20:1) are as safe and potent as pure CBD, this issue should be evaluated in future studies. Notably, artisanal preparations, if used, should be obtained from government-controlled sources (preferably good manufacturing practices-approved) as several studies demonstrated significant inconsistency between product labels and actual content in many cases.

In ASD, the gap between published evidence and public beliefs is much wider. Preclinical studies and case series, reporting successful treatment with artisanal CBD-rich cannabis strains in children with ASD and severe irritability, have triggered widespread use of various cannabis strains in children with ASD, despite a lack of published controlled studies. Moreover, parents often request medical cannabis to treat the core autistic symptoms—not the associated irritability—and this request often comes from parents of children younger than 5 years. Some parents prefer to try medical cannabis for irritability as a first-line treatment, as it is perceived as natural and hence safer, compared with the FDA-approved antipsychotics, risperidone and aripiprazole. Many families are interested in products with a relatively high content of THC, which carries a higher risk of severe neurobehavioral comorbidities in this vulnerable population compared with the general population.

In our opinion, the use of medical cannabis in ASD should be currently limited to clinical trials and highly selected cases of treatment-resistant severe irritability.

## Abbreviations

ASD: autism spectrum disorder
CBD: cannabidiol
THC: Δ9-tetrahydrocannabinol.
